# State-dependent network reconstruction from calcium imaging signals

**DOI:** 10.1186/1471-2202-12-S1-P117

**Published:** 2011-07-18

**Authors:** Olav F Stetter, Demian Battaglia, Jordi Soriano, Theo Geisel

**Affiliations:** 1 Max Planck Institute for Dynamics and Self-Organization, Göttingen, 37073, Germany; 2 Bernstein Center for Computational Neuroscience, Göttingen, 37073, Germany; 3 Universitat de Barcelona, Spain

## 

Calcium imaging has become a standard technique for the measurement of the activity of a population of cultured neurons. Typically these recordings are slow compared to the cell dynamics and display a low signal-to-noise ratio, but they allow for the simultaneous recording of hundreds of neurons.

We are interested in reconstructing an approximation of the structural connectivity of a culture of neurons. This would allow for characterization of the bulk properties of these networks, such as the dependence of connection probability of two nodes on the distance between them, the degree distribution or the clustering coefficient, which are currently inaccessible with single-cell or even typical multi-electrode techniques. In order to benchmark our connectivity inference methods, we first study simulations of fluorescence signals and examine established methods of inferring the topology. It turns out that we can improve on these methods if we turn to measures from information theory, which do not rely on a linearity assumption.

**Figure 1:**
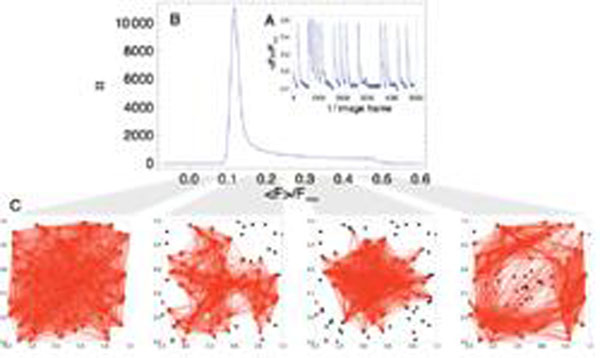
The averaged fluorescence signal of the nodes in our network (A) reveals the existence of quiet phases and network spikes, which is then represented in the histogram over time (B). The effective connectivity calculated from the data points when the averaged signal is in a given interval then depends strongly on this interval, and corresponds best to the actual, locally clustered topology (not shown) is at around 0.2 <F>/F_max_. (C) The links are derived by thresholding the transfer entropy values to the top 20%.

Because we are interested in directed networks, our measure of choice is Transfer Entropy [1,2]. It turns out that we can achieve a high quality of the reconstruction if we allow for novel extensions of this measure. Specifically, we need to take into account the ability of the network to display different dynamical states (fig. 1). We need to focus on phases of activity where the dynamics in the network are dominated by direct monosynaptic interactions, and where therefore the effective connectivity corresponds closely to the structural connectivity. Additionally, we need to correct for the slow acquisition rate of the recording by allowing for instantaneous interactions between nodes in addition to interactions from different image frames.

We demonstrate post-processing improvements of the reconstruction using the Data Processing Inequality that are only possible in the case of information theoretical measures. These methods, already applied with success in the reconstruction of gene regulatory networks [3], help to discriminate indirect from direct interactions.

We then apply our algorithm to real data from large cultures of hippocampal neurons in vitro stained with Fluo-4 AM dye. We probe and quantify the distance-dependent probability of connection and other topological properties of the reconstructed network, finding deviations from a random topology.

Finally we point out and quantify which experimental parameters would be most relevant for an improved reconstruction using our method.
